# Vaccine for Diabetes—Where Do We Stand?

**DOI:** 10.3390/ijms23169470

**Published:** 2022-08-22

**Authors:** Dinesh Kumar Chellappan, Richie R. Bhandare, Afzal B. Shaik, Krishna Prasad, Nurfatihah Azlyna Ahmad Suhaimi, Wei Sheng Yap, Arpita Das, Pradipta Banerjee, Nandini Ghosh, Tanner Guith, Amitava Das, Sarannya Balakrishnan, Mayuren Candasamy, Jayashree Mayuren, Kishneth Palaniveloo, Gaurav Gupta, Sachin Kumar Singh, Kamal Dua

**Affiliations:** 1 Department of Life Sciences, School of Pharmacy, International Medical University, Kuala Lumpur 57000, Malaysia; 2 Department of Pharmaceutical Sciences, College of Pharmacy & Health Sciences, Ajman University, Al-Jruf, Ajman P.O. Box 346, United Arab Emirates; 3 Center of Medical and Bio-Allied Health Sciences Research, Ajman University, Al-Jruf, Ajman P.O. Box 346, United Arab Emirates; 4 St. Mary’s College of Pharmacy, St. Mary’s Group of Institutions Guntur, Chebrolu, Guntur 522212, India; 5 Department of Clinical Sciences, College of Dentistry, Centre of Medical and Bio-Allied Health Science Research, Ajman University, Al-Jruf, Ajman P.O. Box 346, United Arab Emirates; 6 School of Health Sciences, International Medical University, Kuala Lumpur 57000, Malaysia; 7 Department of Biotechnology, Adamas University, Kolkata 700126, India; 8 Department of Surgery, Indiana University School of Medicine, Indianapolis, IN 46202, USA; 9 School of Pharmacy, International Medical University, Kuala Lumpur 57000, Malaysia; 10 Department of Pharmaceutical Technology, School of Pharmacy, International Medical University, Kuala Lumpur 57000, Malaysia; 11 C302, Institute of Ocean and Earth Sciences, University of Malaya, Kuala Lumpur 50603, Malaysia; 12 School of Pharmacy, Suresh Gyan Vihar University, Jaipur 302017, India; 13 Department of Pharmacology, Saveetha Dental College, Saveetha Institute of Medical and Technical Sciences, Saveetha University, Chennai 600077, India; 14 Uttaranchal Institute of Pharmaceutical Sciences, Uttaranchal University, Dehradun 248007, India; 15 School of Pharmaceutical Sciences, Lovely Professional University, Jalandhar-Delhi G.T Road, Phagwara 144411, India; 16 Australian Research Centre in Complementary and Integrative Medicine, Faculty of Health, University of Technology Sydney, Sydney, NSW 2007, Australia; 17 Discipline of Pharmacy, Graduate School of Health, University of Technology Sydney, Sydney, NSW 2007, Australia

**Keywords:** diabetes, vaccines, clinical trials, insulin, GLP

## Abstract

Diabetes is an endocrinological disorder with a rapidly increasing number of patients globally. Over the last few years, the alarming status of diabetes has become a pivotal factor pertaining to morbidity and mortality among the youth as well as middle-aged people. Current developments in our understanding related to autoimmune responses leading to diabetes have developed a cause for concern in the prospective usage of immunomodulatory agents to prevent diabetes. The mechanism of action of vaccines varies greatly, such as removing autoreactive T cells and inhibiting the interactions between immune cells. Currently, most developed diabetes vaccines have been tested in animal models, while only a few human trials have been completed with positive outcomes. In this review, we investigate the undergoing clinical trial studies for the development of a prototype diabetes vaccine.

## 1. Introduction

Persisting as a major global health threat, diabetes mellitus (DM) affects individuals of all ages, ethnicities, and backgrounds, especially those associated with a prominent family history of diabetes and a multitude of environmental factors [1,2,3,4]. As reported by the World Health Organization (WHO), 422 million people globally suffer from diabetes and is rapidly progressing in intermediate and poverty-stricken nations [5]. Approximately 1.5 million deaths annually are caused by diabetes worldwide [5]. It has been reported that China contributed to the highest number of diabetics in 2021, with 149.1 million of its population between ages 20 and 79 being affected by this chronic disease. It is forecasted that China will have approximately 174 million diabetic patients by the year 2045. Meanwhile, a survey in 2014 by Kaveeshwar and Cornwall which reported an elevation approaching 8.3% in diabetic incidences further elucidates this observation [6,7,8]. Complications stemming from poorly managed DM represent a crucial cause of concern as a threat to mortality, indirectly impacting the economical status of a country [9]. The development of secondary complications worsens the mortality and morbidity caused by diabetes [10]. Initially, the classification for diabetes depended on its etiology and clinical course, before ultimately being categorized into Type 1 (T1DM) and Type 2 diabetes (T2DM), as the previous definition excluded many sufferers who exhibited atypical presentation and progression of the disease [11,12]. According to the American Diabetes Association, T1DM occurs due to defects in insulin production, whereas T2DM precipitates primarily from insulin resistance, followed by problematic reduction in insulin secretion, giving rise to hyperglycemia [13,14].

Orban et al. (2001) elucidated three phases whereby researchers may interrupt the underlying pathologic mechanisms behind T1DM, which are the autoimmunity development, autoantibody development, and clinical manifestation emergence with remaining residual β-cell function to be conserved [15]. The intention of halting these phases is to avert autoimmunity development in its initial stages as well as to inhibit clinical disease onset in high-risk individuals, since this phase is the root cause of the disease for the vast majority of patients [16]. However, as for the prevention of T2DM progression, the International Diabetes Federation (IDF), in 2006, proposed a method involving reduction in modifiable risk factors [17,18]. In terms of monitoring parameters, autoantibodies, such as insulin, insulinoma-associated protein 2 (IA-2), glutamic acid decarboxylase (GAD) or zinc transporter isoform 8 (ZT8), act as biomarkers to detect the preliminary onset of diabetes, as individuals who tested positive for more than 50% of these autoantibodies compared to single β-cell antigens are at a greater risk of developing T1DM [19,20]. On the other hand, β-cell destruction is mediated by different types of cytokines or by the direct activity of T- or B lymphocytes.

The pancreatic β-cell damage may be initiated by direct environmental toxins, a virus, or a primary immune attack against pancreatic β-cell antigens such as glutamic acid decarboxylase 65-kD antibody (GAD65). T-helper lymphocytes, such as CD4+, are activated by β-cell antigens and antigen-presenting cells, including the dendritic cells (DC) and macrophages. Interleukin (IL)-12 secreted by macrophages then stimulate the secretion of IL-2 and interferon (IFN)-γ by the CD4+ T-cells. IFN-γ then excites further resting macrophages to secrete other cytokines, such as the tumor necrosis factor (TNF-α), free radicals and IL-1β, which are lethal for pancreatic β-cells. Additionally, activated T-helper cells produce cytokines which attract T- and B lymphocytes and trigger its multiplication in the islet of Langerhans, hence precipitating insulitis. With time, B lymphocytes would attack and harm the β cells by producing antibodies against secreted pancreatic β-cell antigens, whereas cytotoxic T-lymphocytes (CD8+) directly attack β cells which carry the target autoantigens [21,22,23].

As diabetes is a progressive disease, diabetic patients require effective, long-term treatment and the regular monitoring of treatment to achieve the suggested glycemic HbA1c levels. This management strategy may involve a combination of regimens of oral medicines, injectables, such as insulin or GLP-1 analogs, or both dosage forms. These combinational therapies or injectable therapies confer a high chance of inducing side effects, such as diarrhea or vomiting, with GLP-1 analogs and weight gain or hypoglycemia following insulin treatment. Although certain treatment regimens are unsuccessful at decreasing a patients’ HbA1c to the desired level, the undesirable side effects of the medications itself causes patients to skip treatment, especially with higher doses, rendering the therapy ineffective. Presently, a patients’ lack of adherence to their treatment plan remains a persistent clinical challenge, with over 50% of diabetic patients failing to strictly follow schedule of medication administration. In addition, although adherence to insulin treatment has improved in the past few years, due to the usage of pre-mixed formulas and smaller-sized needles, it remains sub-optimal at 63–65% [24,25].

**Figure 1:**
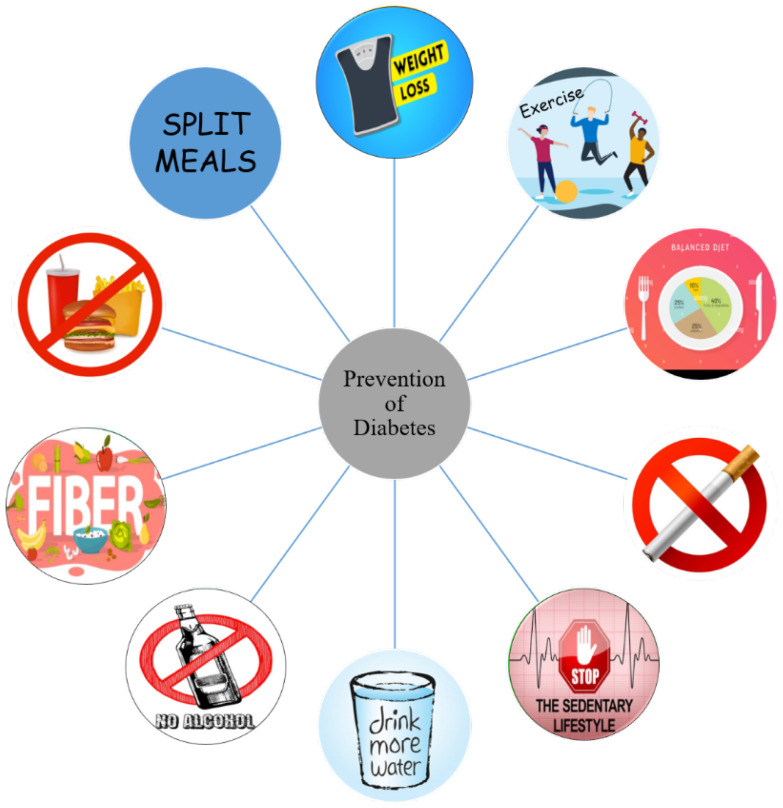
Different modes of prevention of diabetes.

Hence, in an effort to avert medication adherence problems for chronic diseases such as diabetes, there is a growing need for better prevention measures. Recent approaches other than intervening with environmental triggers to halt the onset of DM early has led to the discovery of effective vaccines. In this review article, we attempt to discuss advanced methods of diabetes prevention and the role of adjuvants in relation to vaccines. Some common practices for the prevention of diabetes at early stages is depicted in Figure 1. Ongoing debates and different opinions on various vaccine products made from proteins, antigens, and live pathogens were also examined. Besides that, we also reviewed other types of vaccines from different diseases which may be useful in paving the path for diabetes vaccine development.

## 2. Vaccination

**Figure 2:**
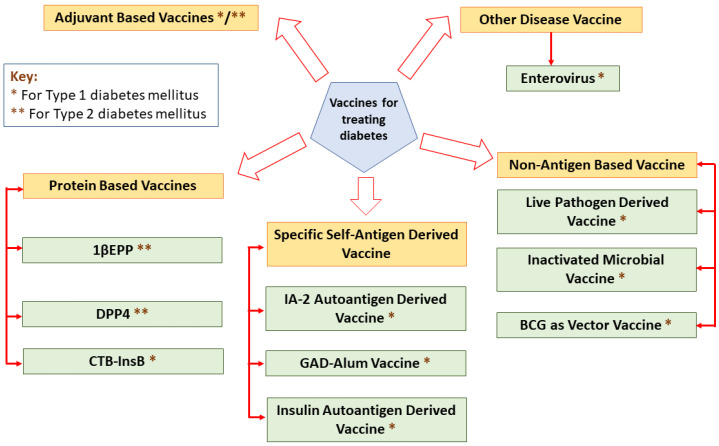
A classification of different vaccines for treating diabetes.

Vaccines elicit their responses in several ways: dampening the destructive Th1 immune response to a benign Th2 response, inciting antigen-specific T-reg cells, eradicating autoreactive T cells or arresting immune cell interaction [26]. A classification of different vaccines for treating diabetes is mentioned in Figure 2.

### 2.1. Early Diabetes Prevention

There are various approaches involved in preventing DM onset and progression, mainly by treating targeted individuals with a family history of diabetes or tenacious autoantibodies, intensive lifestyle interventions, the consumption of dietary fibers and the intake of vitamin D supplements [20,27,28,29,30,31]. However, the possibility of immunization being a method of prevention remains under-researched. At present, vaccines are employed as prophylactic measures in combating infectious diseases by using variants derived mainly from targeted live-attenuated pathogens. However, concerns pertaining to the safety profile of these vaccines has led to the investigation of more advanced bases known as adjuvants, which perform a key role in skewing immune responses and their fabrication [32,33,34].

### 2.2. Rationale behind Vaccine Adjuvant Action

A new era of vaccine development is presently emerging through novel combined therapy comprising adjuvants, which specifically activate and drive immune responses [35,36,37,38]. Traditionally, incorporating an adjuvant into a vaccine presents certain benefits, such as a reduced quantity of dose administered, leading to altered immune responses of greater quality, with minimal side effects [39,40,41]. A prime example of frequently used adjuvants includes alum adjuvants, which are readily available in the market today, as this compound assists in promoting humoral immunity in an individual [42,43].

## 3. Newly Designed Vaccine Products

Currently, several utilized vaccine products, such as autoantigens and non-autoantigen-specific therapies, are underway to be developed into vaccines for diabetes. A few of these have almost reached the final human testing stage. Hence, in this article, we aim to discuss various approaches incorporating prevention strategies with vaccines with respect to diabetes mellitus.

### 3.1. Protein-Based Approach in Vaccine Production

#### 3.1.1. IL-1β-Targeted Epitope Peptide (1βEPP) as a New Vaccine Product for T2DM

Inflammation of the pancreatic islet in T2DM leading to β-cell apoptosis and disruption of insulin production is mainly caused by IL-1β cytokine, a key mediator that induces insulin resistance within the peripheral tissue [44,45,46,47,48]. However, several studies have interestingly shown that IL-1β is capable and has potential to be enhanced as a T2DM future therapy. To elaborate on this revelation, not only did a newly developed IL-1β-targeted epitope peptide vaccine adjuvant with polylactic acid microparticles (1βEPP) stimulate the level of glucose tolerance and provide a hyperglycaemia shield when tested in diabetic KK-Aay mice model, but it also caused a reduction in the lipid profile and β-cell apoptosis action [49]. However, a few alterations were made to create a securely modified anti-IL-1β to address issues derived from the phase I and II trial studies [50]. Another noteworthy research highlighting the use of a combination therapy (CT) of anti-IL-1β and GAD65 DNA vaccine demonstrated the immense potential in reversing diabetes in its early stages [51]. Besides that, another vaccine, hlL1bQb was initially developed and tested in a preclinical simian phase before being tested in T2DM patients, where it was well documented that the hlL1bQb immunization caused harm to human subjects involved in the trial [52].

#### 3.1.2. Dipeptidyl Peptidase-4 Inhibitor (DPP4) as Novel Vaccine Product for T2DM

Incretins are hormones which are secreted from enteroendocrine cells into the blood within minutes after food intake to regulate the amount of insulin to be secreted. Incretins essentially consist of two variants, one being the glucose-dependent insulinotropic peptide (GIP) and another being the glucagon-like peptide-1 (GLP-1). Although these hormones share numerous common actions in the pancreas, they exhibit very distinct actions outside of the pancreas. Since both types of incretins are quickly deactivated by a dipeptidyl peptidase-4 inhibitor (DPP4) enzyme, DPP4 inhibitors, on the other hand, raise the concentrations of these hormones, resulting in enhanced β-cell responsiveness to raised glucose concentrations as well as the suppression of glucagon secretion [53,54]. On the other hand, activation of glucagon-like-peptide (GLP) receptor agonists results in insulin secretion and caspase-mediated cell death inhibition in pancreatic β-cells through the action of DPP4. Earlier researchers recognized GLP and DPP4 as both efficacious and long-lasting agents for future approaches in treating T2DM [55,56,57,58,59,60,61,62], as elucidated by a study performed in 2014 which succeeded in synthesizing a DPP4 vaccine. When trials were performed in C57BL/6J mice model, results revealed a rise in GLP-1 level and an enhanced sensitivity of insulin without prompting adverse autoimmune responses. This occurrence is mainly observed in B- and T-cell epitopes, where a significant increase in anti-DPP4 antibody titre is detected along with the Th2 humoral response [63]. Recently, a study was conducted using D41-IP, a newly combinatorial peptide vaccine, synthesized using the B-cell epitope D41 within DPP4 and B-cell epitope of insulinoma antigen-2 (IA-2) in an effort to enhance therapeutic responses [64,65]. However, this multi-epitope study is perceived more as an alternative diabetic therapy, compared to the previous study, which focused on diabetes onset prevention [65].

#### 3.1.3. CTB-InsB Vaccination Product to Treat T1DM

Bombyx mori, a classic host which secretes recombinant proteins in its fifth instar stage from the lumen of silk glands, has been used in silkworm biotechnology for ages [66,67,68]. This discovery by Dr. Maeda in 1985 originated from silkworm larvae and has drawn much attention due to its high level of recombinant protein expression [69]. Cholera toxin subunit B (CTB), which is known for its toxic characteristics, is usually used as strong adjuvant, which, together with its antigen coupling action, will eventually lead to a down-regulation response in the onset of T1DM [70]. On the other hand, the B chain of insulin (INSB) is briefly known as a 30-amino-acid chain with an immunogenic epitope. When used in combination, both CTB and INSB make up a consumable vaccine to induce immune tolerance in diabetic patients, with its strong influences in evading insulitis when tested in NOD mice. This specific tolerance increases Foxp3+ regulatory T-cell proportions in peripheral lymph tissues and suppresses the biological functions of spleen lymphocytes in mice. This important research proved the effectiveness of the CTB-InsB oral protein vaccine against diabetes development [71,72,73,74,75].

### 3.2. Specific Self-Antigens Approach in Vaccine Production

**Figure 3:**
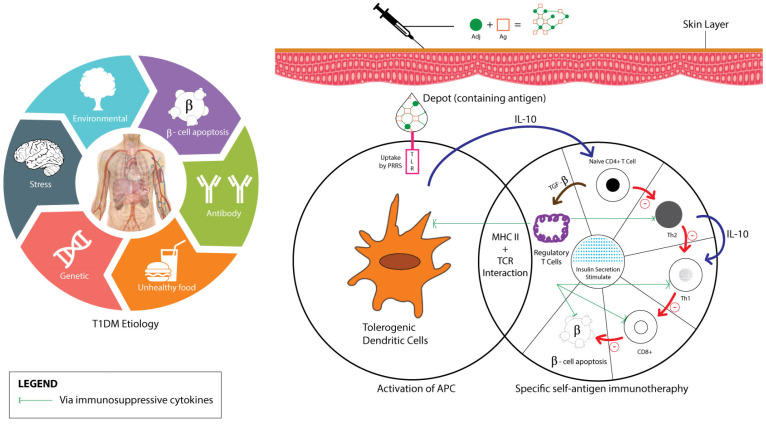
T1DM and vaccine adjuvant’s mechanisms of action.

Upon administration and absorption of the vaccine-adjuvant into the T1DM patients’ skin, toll-like-receptor (TLR) acts on depot-containing antigens through pattern recognition receptors (PRRs) activation, which mainly leads to antigen presenting cells (APC) maturation (primarily DC). Activated APCs on major histocompatibility complexes (MHC) surface then interact with antigen-specific T cells and secrete IL-10 cytokine to suppress Th1 by Th0 stimulation. Two pathways—the Th2 anti-inflammatory process and Treg cells induction that suppresses Th cells development—eventually stimulate insulin secretion as diagrammatically shown in Figure 3 [36,76,77,78].

#### 3.2.1. IA-2 as New Vaccine Product for T1DM

The pharmacological action of the D41-IP vaccine in using IA-2 protein as an islet autoantigen has been tested in few different studies [79]. IA-2, a tyrosine phosphatase protein, is commonly known as a T1DM major islet antigen [80]. A study conducted in 2012 by Guan et al. claimed that the IA-2 vaccination is capable of delaying the onset and the late stages of autoimmune diabetes either on its own or when co-administered with plasmid IL-4/MCP-1, which proposed a promising future for T1DM patients [81,82]. Later, a newly designed novel peptide vaccine, IA-2-P2, was introduced to the public, where the overall idea of the vaccine was initiated based on the previous findings of Guan et al. A drastic drop in the blood glucose levels of normoglycemic mice were obtained when tested with the IA-2-P2 vaccine in comparison to P277 and control mice in the study. Hence, it was concluded that IA-2-P2 was a suitable ameliorate vaccine to combat T1DM. The P277 peptide, a human 60 kDa heat shock protein (hsp60) is a causative factor for the onset of diabetes of non-obese diabetic (NOD) mice which are genetically prone to developing spontaneous autoimmune diabetes [83]. However, Lu et al. reported that the fusion of the His-Hsp65-6IA2P2 protein vaccine through nasal inoculation is believed to serve well in regulating the T1DM response [84]. Apart from IA-2, the zinc transporter (ZnT8) has been also studied as a major autoantigen target in T1DM immunotherapy. However, no investigations have been conducted to test ZnT8 as a diabetes vaccine in trials [85,86,87,88].

#### 3.2.2. Glutamic Acid Decarboxylase 65-kD (GAD65): A New Vaccine Product for T1DM

The GAD65 antibody is an isoform of GAD targeted by self-reactive T cells that exhibits susceptibility marker detection in T1DM more frequently as compared to the IA-2 autoantigen [89,90]. On the other hand, aluminum hydroxide is the most used adjuvant [91,92]. In 2011, the GAD-alum vaccine (Diamyd) comprising GAD and aluminum hydroxide, was introduced, in which the efficacy and safety of the vaccine was tested in phase II trials preceding four years of close pharmacovigilance [93,94,95,96,97,98,99]. However, no desirable effects were observed within T1DM subjects, although two to three drops of injected vaccine were used in each subject throughout the three trial stages of experiment, rendering it ineffective [100,101,102]. In fact, HbA1c and insulin were not altered by the GAD-alum treatment [100]. In addition to that, a review article by Cook et al. also highlighted the insufficiency of the GAD-alum vaccine when tested in clinical trials of larger sample sizes [103]. However, the combination of CTB-insulin and CTB-GAD with IL-10 as a newly proposed multi-component vaccine has proven to suppress β-cell autoreactivity in T1DM [104]. Other studies also revealed that GAD65 antibodies elicit activity against glial fibrillary acidic protein (GFAP), a predictive biomarker that is expressed within peri-islet Schwann cells in the event of the onset of T1DM, making it a suitable molecule to be incorporated in the production of an immune tolerizing vaccine [105,106]. Hyperglycemia was suppressed, whereas C-peptide secretion was enhanced significantly in T1DM using this GFAP vaccine by acting upon T-cell entrance into pancreatic islets, which subsequently shifts T-cell differentiation from a cytotoxic Th1- to a Th2-biased humoral response in NOD mice [107]. Considering the complex mechanism of the action of self-antigens toward the immune system, synthetic materials were used instead in one study to co-deliver the immunomodulatory signals, where fabricated microparticle (MP) vaccines were recently established via in vivo and in vitro methods. Therefore, the first biomaterial-based vaccine product, hydrogel/microparticle, was introduced by using dual subcutaneous immunization, and subsequently, a revised version involving the delivery of three shots of the vaccine into NOD mice models was tested [108,109]. On the other hand, Phillips et al. successfully created the first antisense oligonucleotide-formulated microsphere vaccine capable of suppressing diabetes and boosting Foxp3+ T-reg cells without inducing any unfavorable responses [110,111]. Multiple benefits were observed from these autoantigen-specific interventions over those involving nonspecific immune suppression as an immune tolerance therapy, including the reduction in pathogenic peptide epitope response and any related side effects [112]. Even so, it is unable to be predicted if similar favorable outcomes may be obtained from the phase III clinical trial [113]. In a recent double-blinded, randomized, placebo-controlled Phase IIb clinical trial, the intra-lymphatic administration of GAD-alum with vitamin D supplementation appears to preserve C-peptide in patients with recent-onset T1D carrying HLA DR3-DQ2 [114].

#### 3.2.3. Insulin as a Target in New Vaccine Product for T1DM

The BHT-3021 vaccine, which is made of proinsulin, a precursor insulin prohormone, was proven effective in enhancing insulin production within its early developmental stage [115]. Longer-term research for this vaccine was recently announced by NHS Choice in which larger group of 200 participants will be involved. In another study, the reaction between insulin-like growth factor 2 (IGF-2), an insulin dominant self-antigen, and an insulin autoantigen initiated a response resembling negative/tolerogenic self-vaccination, indicating a possible cure for T1DM, as reported by Chentoufi [116]. To further consolidate this revelation, other studies have proven that self-antigen vaccination is one of the most secure strategies against autoimmune diabetes [117].

### 3.3. Non-Antigen Specific Approach in Vaccine Production

Certain immunologically active microbes and their products have been reported to prevent autoimmune diabetes in different animal models [118]. These agents may confer a protective effect in humans by stimulating the immune system especially during childhood development [118]. As per “the hygiene hypothesis,” the growing cases of autoimmune diseases may be caused by insufficient microbial exposure due to the improved hygienic conditions of the developed world [118]. Some of these microbial approaches are discussed below.

#### 3.3.1. Live Pathogen *Salmonella* as Vector Vaccine

Live recombinant attenuated *Salmonella*-vectored vaccines exhibit great potential as resources to improve human health by achieving long-lasting mucosal, humoral and cellular immunity against a variety of non-*Salmonella* pathogens at a low cost. The use of recombinant DNA has been a major breakthrough in antigen mucosal delivery for years through the generation of a live attenuated *Salmonella* oral vaccine since it was initially tested in phase I clinical trials [119]. It is the T-cell autoreactive downregulation response in the *Salmonella* vaccine which conferred greater therapeutic effectiveness besides its simplicity and relative safety in comparison to antigen-specific approaches, thus making it a potential future therapy for T1DM despite an uncertain mechanism of action [120]. A current study utilized *Salmonella typhimurium* bacteria in combination with other small regulatory proteins called cytokines and a low dose of an immunosuppressive drug, Anti-CD3. Results revealed that the vaccine reinstates balance to the immune system and prevents the attack of insulin-producing cells.

#### 3.3.2. Inactivated Microbial Vaccines

Apart from alum and CTB adjuvants, complete Freund’s adjuvant (CFA) has also been studied as a vital T-regulatory cell inducer which successfully reverses autoimmune diabetes through multicomponent immunization. However, the injection of a combined anti-CXCL10 vaccine with CFA is not advisable for human use due to its high toxicity profile, despite its proven effectiveness, as elucidated in a study by Oikawa et al. (2010), whereby the anti-CXCL10 vaccine used successfully reversed T1DM [121]. In few years, further studies involving CFA were performed using CTB:GAD fusion protein, which successfully induced the protective effects in tested NOD mice [122]. In more recent times, a multivalent islet lysate-negative vaccine tested elicited a positive immunogenic response in the pathophysiology of diabetes, in which incomplete Freund’s adjuvant (IFA) was used [123].

#### 3.3.3. BCG as Vector Vaccine in Clinical Trial Studies

In 2001, tumor necrosis factor (TNF-α) activation following BCG vaccine administration to restore endogenous β-cell function resulted in the discovery of a potential T1DM reversal mechanism [124]. In addition to that, Faustman (2012) developed another BCG vaccine which was tested in a phase I randomized control trial, resulting in a proven ability of the BCG vaccine in triggering TNF to induce apoptosis in autoreactive T cells. Furthermore, an increase in the restoration rate of pancreatic β-cell function was observed in a rodent model response [125]. Likewise, a minimum of two doses of the BCG vaccine is recommended, particularly with the first dose being administered in neonates. However, further studies pertaining to this claim is required, as its mechanism of action in human subjects remains unclear [126,127]. Nevertheless, no definite linkage was found in the preservation of β-cell function in T1DM after prophylactic administration of the BCG as hypothesized by other randomized clinical studies between the years 1999 and 2005 [128]. In contrast, approval by the Food and Drug Administration (FDA) led to the commencement of a phase II clinical trial study regarding the activity of the BCG vaccine related to T1DM reversal. This study design involves 130 participants, of which approximately over 100 candidates have received a minimum of one dose of the BCG vaccine, and progress or response is actively being monitored for five years from the date of administration.

## 4. Potential of Other Disease-Vaccines in Treating Diabetes

Interestingly, previous research elucidated evidence for enteroviruses (EV) being a causative factor for the onset of type I diabetes. Hence, avid and ongoing effort in conducting research and development to develop and synthesize an EV vaccine is currently in progress with the aid of technological advances. Yet, various issues are still under discussion, such as diabetogenic EV serotypes, safety concerns pertaining to it, and relevance as well as accuracy of the accumulated literature reviews, before this novel vaccine can be tested in clinical trials [129,130]. However, a noteworthy accomplishment was achieved in a recent preclinical study involving the invention of the first multivalent formalin inactivated CVB1 vaccine, where the vaccine proved to be effective with no adverse effects [131]. Apart from the usage of vaccines indicated for viral infections, the tuberculosis DNA vaccine known as DNA-HSP65 exemplifies high possibility in becoming the latest immunotherapeutic agent for the management of diabetes [132]. A list of new vaccine products in the pipeline that could be employed for diabetes can be found in Table 1.

### Future Perspectives

Despite various prospects and the effectiveness of each approach discussed previously, limitations which arise should be thoroughly considered and evaluated from various perspectives, where modifications may be made for future immunotherapies and later implemented in phase III and IV trials. To date, the majority of the diabetes vaccine development has been studied using animal models, with relatively few human trials [26]. A major limitation of using animal models, such as NOD mice, is their profound sensitivity to diabetes protection. As a result, several successful animal studies failed in human trials, including the Diabetes Prevention Trial-1 [26]. Recently studies have suggested the administration of non-depleting anti-CD3 antibodies or a peptide from heat shock protein 60 to be beneficial against a recent onset of T1DM [26]. The revolutionary Diamyd vaccine for the prevention of diabetes, for example, could possibly be improved via the exploration of other antigen delivery pathways, such as self-antigen DNA vaccine administration, with consideration of divergent dose administration or replacement with a different type of autoantigen [133,134]. Besides that, understanding various other routes of administration could potentially be an area of further research. Being another pivotal part of vaccine development, feasible study designs not only contribute toward grasping a better understanding of the Alum-GAD system but also could pave the path to develop newer combination therapies studies for diabetes.

## 5. Conclusions

The pressing concern pertaining to the rising number of patients falling victim to diabetes has garnered the interest of numerous scientists and researchers worldwide in the search for the most effective management strategies, as demonstrated with the abundance of literature available to date. The urgent need for more effective prophylaxis in addition to conventional dietary modification advice to patients has prompted various attempts in developing vaccines to delay or prevent the onset of this chronic disease, as seen in recent approaches where protein-based, self-antigen and non-antigen specific interventions have exhibited promising potential for use as vaccines against diabetes in the near future. However, a deeper understanding is still required to ameliorate and invent potent therapies with minimal side effects regardless of the cause-related factors, especially for chronic diseases, such as T2DM.

## Figures and Tables

**Table 1:** List of vaccine products for diabetes.

Type of Vaccine	Vaccine Name	Indication	Mechanism of Action	References
Adjuvant-based vaccines	Alum adjuvant-based vaccine	T1DM and T2DM	Activation of immune response; Promoting humoral immunity	[42,43]
Protein-based vaccines	IL-1β-targeted epitope peptide (1βEPP)	T2DM	Alters the level of glucose tolerance and provides a hyperglycaemia shield	[49]
	Dipeptidyl peptidase-4 inhibitor (DPP4) based vaccine	T2DM	Inhibition of dipeptidyl peptidase-4 inhibitor (DPP4) enzyme	[53,54]
	CTB-InsB vaccination product	T1DM	Down-regulation response in the onset of T1DM; Induction of immune tolerance	[71,72,73,74,75]
Specific self-antigen-based approach in vaccine production	IA-2 as a vaccine product	T1DM	Islet autoantigen mechanism; delaying the onset and the late stages of autoimmune diabetes	[81,82]
	Glutamic Acid Decarboxylase 65-kD (GAD65)-Alum vaccine	T1DM	Suppression of β-cell autoreactivity	[104]
	Insulin autoantigen-based vaccine	T1DM	Enhancing insulin production	[115]
Non-Antigen-based vaccines	Live pathogen Salmonella-based vector vaccine	T1DM	T-cell autoreactive downregulation response	[119]
	Inactivated microbial vaccine	T1DM	Positive immunogenic response induction	[121]
	BCG as a vector vaccine	T1DM	Restoration of endogenous β-cell function	[124]
Other Disease-Based Vaccines	Enteroviruses (EV)-based vaccine	T1DM	Delayed onset response	[129,130]

## Data Availability

Not applicable.

## Abbreviations

The following abbreviations are used in this manuscript:

1βEPP	IL-1β-targeted epitope peptide vaccine adjuvant with polylactic acid microparticles
ADA	American Diabetes Association
Alum	Aluminum
APC	Antigen presenting cells
BCG	Bacillus Calmette–Guerin
CFA	Complete Freund’s adjuvant
CTB	Cholera toxin B subunit
DC	Dendritic cell
DM	Diabetes mellitus
DNA	Deoxyribonucleic acid
DPP4	Dipeptidyl-peptidase-4-inhibitor
EV	Enteroviruses
FDA	Food and Drug Administration
GAD65	Glutamic acid decarboxylase 65-kD antibody
GFAP	Glial fibrillary acidic protein
GLP	Glucagon-like-peptide
IA-2	Insulinoma-associated protein 2
IDF	International Diabetes Federation
IFA	Incomplete Freund’s adjuvant
INSB	B chain of insulin
IL-1β	Interleukin-1β cytokine
IL-10	Interleukin-10
MHC	Major histocompatibility complexes
MP	Microparticle
NOD	Non-obese diabetic
PRR	Pattern recognition receptors
T1DM	Type 1 diabetes mellitus
T2DM	Type 2 diabetes mellitus
Th0	Naive helper T cell
Th1	Cytotoxic helper T cell
Th2	Humoral helper T cell
TLR	Toll-like receptor
TNF	Tumor necrosis factor
Treg cells	Regulatory T cells
WHO	World Health Organization
ZnT8	Zinc transporter isoform 8
